# Erastin-induced ferroptosis is a regulator for the growth and function of human pancreatic islet-like cell clusters

**DOI:** 10.1186/s13619-020-00055-3

**Published:** 2020-09-07

**Authors:** Xing Yu Li, Po Sing Leung

**Affiliations:** 1 School of Biomedical Sciences, Faculty of Medicine, The Chinese University of Hong Kong, Shatin, Hong Kong China; 2 Key Laboratory for Regenerative Medicine, Ministry of Education, School of Biomedical Sciences, Faculty of Medicine, The Chinese University of Hong Kong, Hong Kong, China

**Keywords:** Apoptosis, Autophagy, Cell death, Erastin, Ferrostatin-1, Islets, NOX4, p38

## Abstract

Ferroptosis is a newly identified and novel form of cell death, which is characterized by an iron- and reactive oxygen species (ROS)-dependent manner. Potential utility of ferroptotic cell death has been recently proposed for cancer treatment. Meanwhile, ROS generation and apoptosis are inherently consequent to cell apoptosis and dysfunction during islet cell preparation and transplantation. Whether ferroptosis induction is a regulator for cell viability and function in human pancreatic islet-cell clusters (ICCs) derived from pancreatic progenitor cells (PPCs) remains elusive. We thus sought to induce ferroptosis in our established cell culture system of human PPCs/ICCs, examine the effects of ferroptosis on ICCs, and explore the potential regulatory pathways involved. Our results showed that ICCs were prone to the use of ferroptosis-inducing and inhibiting agents under our culture conditions. Erastin, a ferroptosis inducer, was found to trigger ferroptosis in ICCs, without the apparent detection of other types of cell death involved, such as apoptosis and autophagy. In corroboration, the use of ferroptosis inhibitor, ferrostatin-1 (Fer-1), was found to enhance the cell viability of ICCs and prevent them from ferroptosis as well as improve its function. Mechanistically, the erastin-induced ferroptosis in ICCs was probably mediated via activation of JNK/P38/MAPK pathways and upregulation of NOX4 expression. Together, our findings may provide a scientific basis of ferroptosis inhibition as a potential for the amelioration of ICC survival and functionality during islet transplantation in diabetic patients.

## Background

Ferroptosis was first identified in 2012 as a novel form of cell death, which was characterized by an iron- and reactive oxygen species (ROS)- dependent manner (Dixon et al. 2012). Long before the identification of ferroptosis, there were reports revealing an oncogenic RAS-related cell death induced by two drugs, named erastin and RSL-3 (Dolma et al. 2003; Yang 2008). This newly identified type of cell death is distinct from the classical cell death in many ways, and thus implications of its role in heath and diseases are ensured. As ferroptosis is an iron-dependent cell death, it can be interfered by the administration of iron chelators or endogenous irons in cells (Dolma et al. 2003; Yagoda et al. 2007). The trigger of this iron-dependent cell death is peroxidation-driven so that our investigation into the response to ROS is of clinical relevance (Dixon et al. 2012; Yang et al. 2014; Yang et al. 2016; Kagan et al. 2017). In vivo inactivation of glutathione peroxidase 4 (GPX4) results in the abnormalities in lipid peroxides metabolism, thereby leading to ferroptotic cell death (Stockwell 2018). Another cause of ferroptosis is the abnormal production of lipid hydroperoxides from arachidonic acid. Ferroptosis has distinct characteristics that are different from other types of cell death, such as apoptosis and autophagy; they include, but not limited to, the biochemical and morphological features as well as the expression of key genes and regulatory pathways (Mou et al. 2019). Interestingly, this ferroptotic cell death can be induced by various physiological and pathophysiological conditions, notably in different types of cancer cells (Mou et al. 2019; Dixon 2017). Meanwhile, it can also be induced and blocked by a number of small molecules or drugs, e.g. erastin and ferrostatin-1 as an inducer and inhibitor, respectively (Cao and Dixon 2016). In fact, erastin and ferrostatin-1 were employed for ferroptosis induction and inhibition in the context of various cancer studies, including the hepatocellular carcinoma cells and pancreatic cancer cells (Bai et al. 2017; Shintoku et al. 2017). Notwithstanding the existence of these findings, the opportunities and challenges of ferroptosis in pancreatic islet cell survival and function in diabetic medicine have yet to be explored, in particular reference to the clinical potential and roles of ferroptosis inducers and inhibitors in the regulation of transplantable islets during islet cell transplantation.

The major hindrance to the application of clinical islet transplantation is the inherent lack of human cadaveric donors in concert with the inevitable cytotoxic conditions during the islet isolation process and pre−/post- transplantation period; the latter is attributable to cell death cascades, apoptosis and necrosis, thus leading to the loss of transplantable islets and graft functionality (Ryan et al. 2005; Davalli et al. 1996; Xu et al. 2019). To address this issue, the leverage of pancreatic stem cells/progenitor cells provides a platform for stem cell-based research and thus holds a great promise for clinical islet transplantation (Leung and Ng 2013). In this respect, we have recently established a culture system of human pancreatic progenitor cells (PPCs) and islet-like cell clusters (ICCs) derived from human fetal pancreas; in this in vitro cell system, the PPCs is able to undergo proliferation and differentiation into transplantable ICCs in exposure to adequate growth factors and differentiation cocktail or microenvironment (Leung et al. 2012; Wu et al. 2019; Li et al. 2019).

It has been known that the manipulation of various regulated cell death, including ferroptosis, might have profound impact on islet cell survival and cell function during islet transplantation (Bruni et al. 2018a). Of great interest in this context is the recent report showing that islets are indeed susceptible to ferroptosis induction with compromised in vitro human islet viability and function (Bruni et al. 2018b). Indeed, abnormalities in iron metabolism are closely associated with beta-cell dysfunction and death, as clinically manifested by the bronze diabetes in hereditary hemochromatosis (Wiley 2016; Yen et al. 2006) and diabetes in Friedreich’s ataxia (Cnop et al. 2012; Igoillo-Esteve et al. 2020). Whether iron-dependent ferroptosis induction is a regulator for human ICCs with its transplantation potential has yet to be fully explored. In this study, we thus attempted to induce ferroptosis in our human PPCs/ICCs system in order to examine the effects of ferroptosis on the viability and function of ICCs as well as explore the potential underlying pathways involved in this study.

## Methods

### Cell culture of PPCs and ICCs

The preparation of human fetal PPCs and differentiation of PPCs into ICCs was procured as we described previously (Leung et al. 2012; Wu et al. 2019; Li et al. 2019; Liang et al. 2016). Briefly, PPCs of 12-week gestation from passage number under 10 were used in all experiments and they were trypsinized and suspended in ultra-low attachment plates (Corning) for 8-day differentiation into ICCs. PCCs differentiation was initiated in a serum-free DMEM/F12 medium, supplemented with 1× B27, 0.05% bovine serum albumin (BSA), and the cocktail with growth factors consisting of 10 ng/mL hepatic growth factor (HGF), 10 nM exendin-4, and 500 pM betacellulin. The cocktail for directed differentiation of PPCs was conducted as follows: PPCs were harvested by Tryple Select (Invitrogen) and cultured in ultralow attachment plate (Corning) with differentiation cocktail medium containing growth factors. The media were changed every other day for 8 days to allow ICCs formation. Consents for using human fetal pancreas were approved by the Clinical Research Ethics Committee and agreed by donors from the Prince of Wales Hospital of The Chinese University of Hong Kong. Human ethics approval of using human fetal tissues were obtained from the Joint Chinese University of Hong Kong-New Territories East Cluster Clinical Research Ethnics Committee (CREC-2010.574 and CRE-2011.383).

### Measurement of LDH release and cytotoxicity

ICCs were hand picked and challenged with or without the drugs, erastin (20–50 μM) and Fer-1 (1–10 μM) for 24 h. The sample were centrifuged at 4 °C for 250 g × 10 min, then the supertanants were harvested without disturbing the cell pellets. The non-treatment group was used as low control representing the spontaneous LDH release; the samples were lysed by 5% Triton-X and used as high control representing maximal LDH release (Bruni et al. 2018a). The Cytotoxicity Detection Kit (Roche) was used to assess the LDH release according to the manufacturer’s protocol. The calculation of LDH was based on the formula below:

$$ LDH\  release=\frac{test\  LDH\  release- spontaneous\  LDH\  release}{maximal\  LDH\  release} $$

### Measurement of insulin content

Insulin of ICCs was extracted using acid ethanol (0.18 M HCl in 96% ethanol (vol/vol)) at 4 °C overnight as we described previously (Leung et al. 2012), and the concentration was measured by Ultra-sensitive Human Insulin Immunoassay Kit (The University of Hong Kong, Hong Kong, China) according to the manufacturer’s protocol.

### Western blot analysis

Total proteins of ICCs were extracted by CytoBuster protein extraction reagent (Novagen) and diluted by 2 × Laemmli Sample Buffer (Bio-Rad, USA) supplemented with 5% beta-mercaptoethanol and denatured under 100 °C for 5 min as we described previously (Leung et al. 2012; Wu et al. 2019). The samples were then fractionated by SDS/PAGE and transferred to PVDF membranes (Bio-Rad). Membranes were blocked with 5% non-fat milk in phosphate buffered saline with 0.1% Tween-20 (PBST) at 25 °C for 60 min, probed with primary antibodies and then normalized by β-actin at 4 °C overnight. The membranes were rinsed with PBST for 10 min × 3 times and incubated with secondary antibody at 25 °C for 60 min. After rinsed with PBST for 10 min × 3 times, the probed proteins were detected by ECL (GE Healthcare, Piscataway, NJ, USA) on LucentBlue X-ray film (Advansta, USA). Results were analyzed via image J software.

### Analysis of mRNA levels by quantitative real-time PCR

Total RNAs of the ICCs were collected and subjected to reverse transcription. In brief, cells were harvested with TRIzol (Invitrogen, USA) and extracted by chloroform (200 μl/1 ml TRLzol) thoroughly, following the centrifugation with 12,000 g at 4 °C for 15 min. The supernatant was then carefully removed and mixed with isopropanol (200 μl/ml TRLzol), after the centrifugation again at 4 °C for 15 min. The supernatant was discarded and the pellet was washed twice with 75% (v/v) ethanol. Finally, the RNA pellet was resuspended with ultra-pure water (Takara Bio, Japan) and 1–2 μg of RNAs was used for the reverse transcription with PrimeScript RT Master Mix Kit (Takara Bio, Japan). Resultant cDNAs were analyzed by ViiA 7 Real-Time PCR System (Applied Biosystems Life Technologies, U.S.A.). Gene expression levels normalized to β-actin were calculated by comparative threshold cycle method (2^−ΔΔ^Ct) (Leung et al. 2012; Liang et al. 2016). The design of primers was done based on the NCBI Primer-BLAST online tool (https://www.ncbi.nlm.nih.gov/tools/primer-blast/).

### Erastin, Fer-1, and SP600125

The ferroptosis inducer, Erastin, and inhibitor, Fer-1 (Sigma, Oakville, ON) were prepared by dissolving the drugs in dimethylsulfoxide (DMSO) at a stock concentration of 20 mM. The JNK inhibitor, SP600125 (Sigma, USA) was prepared by dissolving the drug in DMSO at a stock concentration of 25 mM. For longterm storage, both reagents were stored at − 20 °C. The drugs used were of high potency and specificity with a range of μM.

### Statistical analysis

Three sets of samples were employed and each set of ICCs was performed 3–5 times independently. Data are expressed as means ± SEM. Analysis was conducted in GraphPad Prism. One-way analysis of variance followed by Tukey’ or Student’s t test were applied to comparation. *P* < 0.05 was considered statistically significant.

## Results

### The viability of ICCs is susceptible to ferroptosis induction

**Fig. 1:**
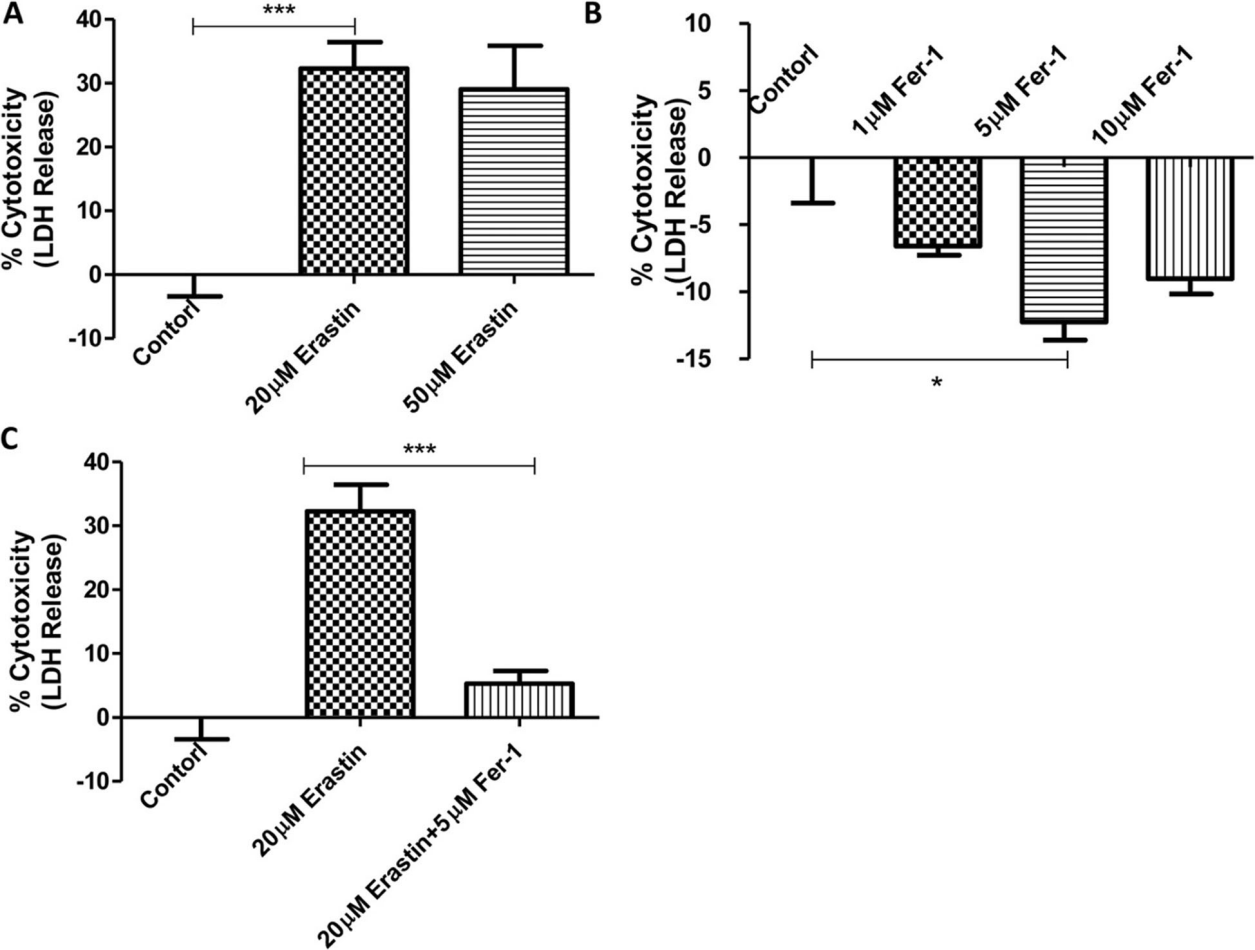
Susceptibility of ICCs to cytotoxicity in term of LDH release with the treatment of ferroptosis inducer, erastin and inhibitor, ferrostatin-1 (Fer-1). **a** ICCs were challenged with 20 μM or 50 μM erastin for 24 h. **b** ICCs were administrated with 1 μM, 5 μM or 10 μM Fer-1 for 24 h. **c** ICCs were treated with 20 μM erastin in the presence or absence of 5 μM Fer-1 for 24 h. The cell free supernatants were harvested for the measurement of LDH release and the viability of ICCs was expressed in percent of cytotoxicity. The experimental groups were analyzed and compared to the respective non-treatment controls. (*n* = 5 per group; *p < 0.05, **p < 0.01, ***p < 0.001. All data are expressed as means ± SEM)

As a first step, we employed the ferroptosis inducer and inhibitor, i.e. erastin and ferrostatin-1 (Fer-1), respectively, to examine and compare the effects of ferroptosis on ICCs. To achieve this, the release level of lactate dehydrogenase (LDH) was measured as a biomarker for the cytotoxicity of ICCs viability compared to the untreated control group. As expected, our results showed that the erastin significantly induced the cell death of ICCs, as evidenced by the increased LDH release in term of percentage of cytotoxicity (Fig. 1 a). On the contrary, the application of Fer-1 significantly improved the viability of ICCs (Fig. 1 b) as well as rescued the cell death induced by erastin (Fig. 1 c), as demonstrated by the assay of LDH release. These data indicate that ICCs viability appeared to be prone to the process of ferroptosis.

### Ferroptosis induction attenuates the insulin-producing ability as well as the expression of differentiation and maturation gene markers of ICCs

**Fig. 2:**
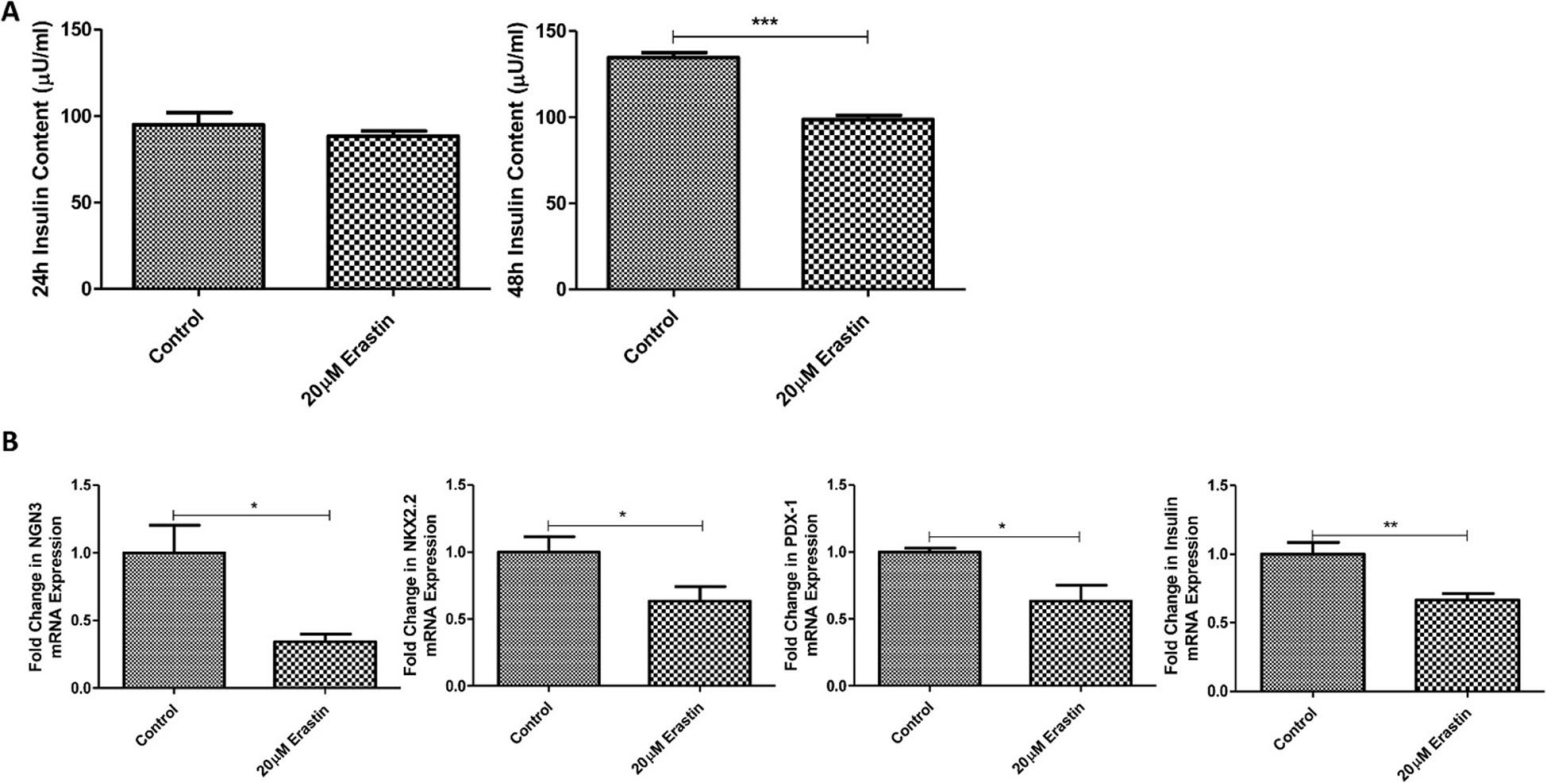
Decreases in insulin content and expression of differentiation/maturation related genes in ICCs under ferroptosis induction. **a** Insulin content of ICCs was performed by Ultra-sensitive Insulin Elisa kit after 24 h or 48 h treatment with 20 μM erastin. **b** Gene expression of differentiation and maturation markers in ICCs (NGN3, NKX2.2, PDX-1 and Insulin) was measured by real-time PCR in relation with the differentiation cocktail derived ICCs. (*n* = 3 per group; *p < 0.05, **p < 0.01, ***p < 0.001. All data are expressed as means ± SEM)

Next, we attempted to examine the effect of ferroptosis on the insulin secretory function of ICCs. As seen, the insulin content of ICCs was observed to be decreased after treatment with 20 μM erastin for 48 h, whereas its treatment with 24 h did not have significant effect on the insulin content (Fig. 2a). Consistently, the expression levels of the differentiation and maturation gene markers of iccs were significantly downregulated after 48 h treatment with erastin; they include NGN3, NKX2.2, PDX-1 and Insulin, relative to their respective controls (Fig. 2b). These data indicate that the erastin-induced ferroptosis was accountable for the reduction in the expression of differentiation/maturation markers as well as insulin synthesis/production of ICCs.

### MAPK pathway is required for the ferroptosis of ICCs

**Fig. 3:**
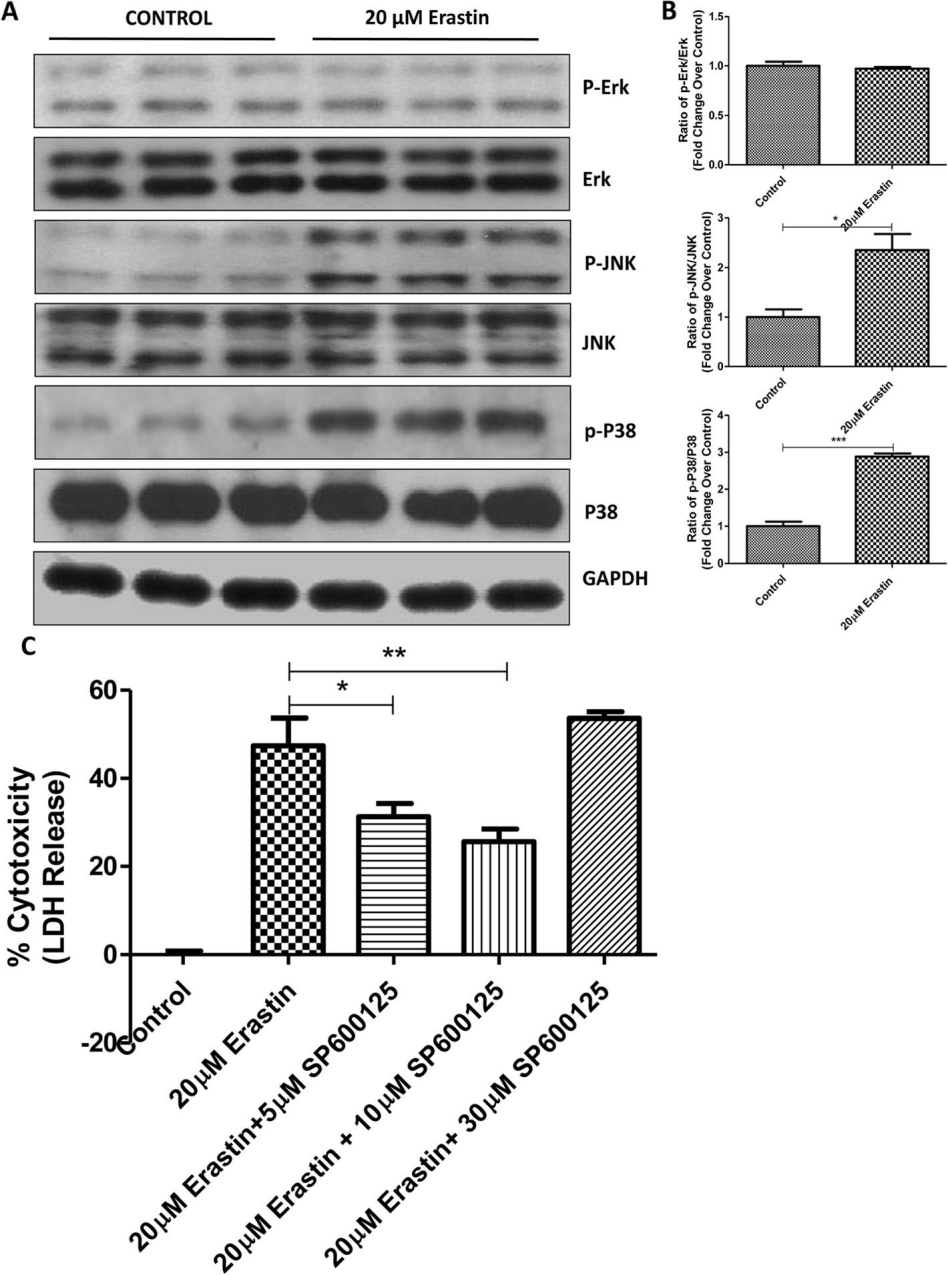
Involvement of MAPK pathway in the induction of ferroptosis in ICCs. ICCs were exposed to 20 μM erastin for 24 h. The expression levels of unphosphorylated and phosphorylated of Erk, JNK and P38 were detected with images (**a**) and quantification (**b**) by Western blot analysis. **c** ICCs were challenged by 20 μM erastin with or without SP600125 (5 μM, 10 μM and 30 μM) for 24 h and the cell free supernatants were harvested for the measurement of LDH release. The data were compared with the non-treatment control group. (*n* = 3 per group; *p < 0.05, **p < 0.01, ***p < 0.001. All data are expressed as means ± SEM)

We then proceeded to explore the potential signaling pathways involved in the ferroptosis in ICCs. As reported, the MAPK pathway was responsive to various stress conditions, including ferroptosis. In this sense, our results revealed that both of the phosphorylated JNK and P38 signaling proteins were upregulated in ICCs by erastin-induced ferroptosis, whereas the ERK protein levels remained unchanged (Fig. 3a & b). To further validate the potential role of MAPK pathway involved in the ferroptosis of ICCs, the JNK inhibitor, SP600125, was employed to counteract the ferroptotic cell death. Our data showed that LDH release or cytotoxicity was markedly reduced in response to the administration of erastin, suggesting the involvement of MAPK-mediated ICCs viability (Fig. 3c).

### Ferroptosis induction upregulates the NOX4 gene expression of ICCs

**Fig. 4:**
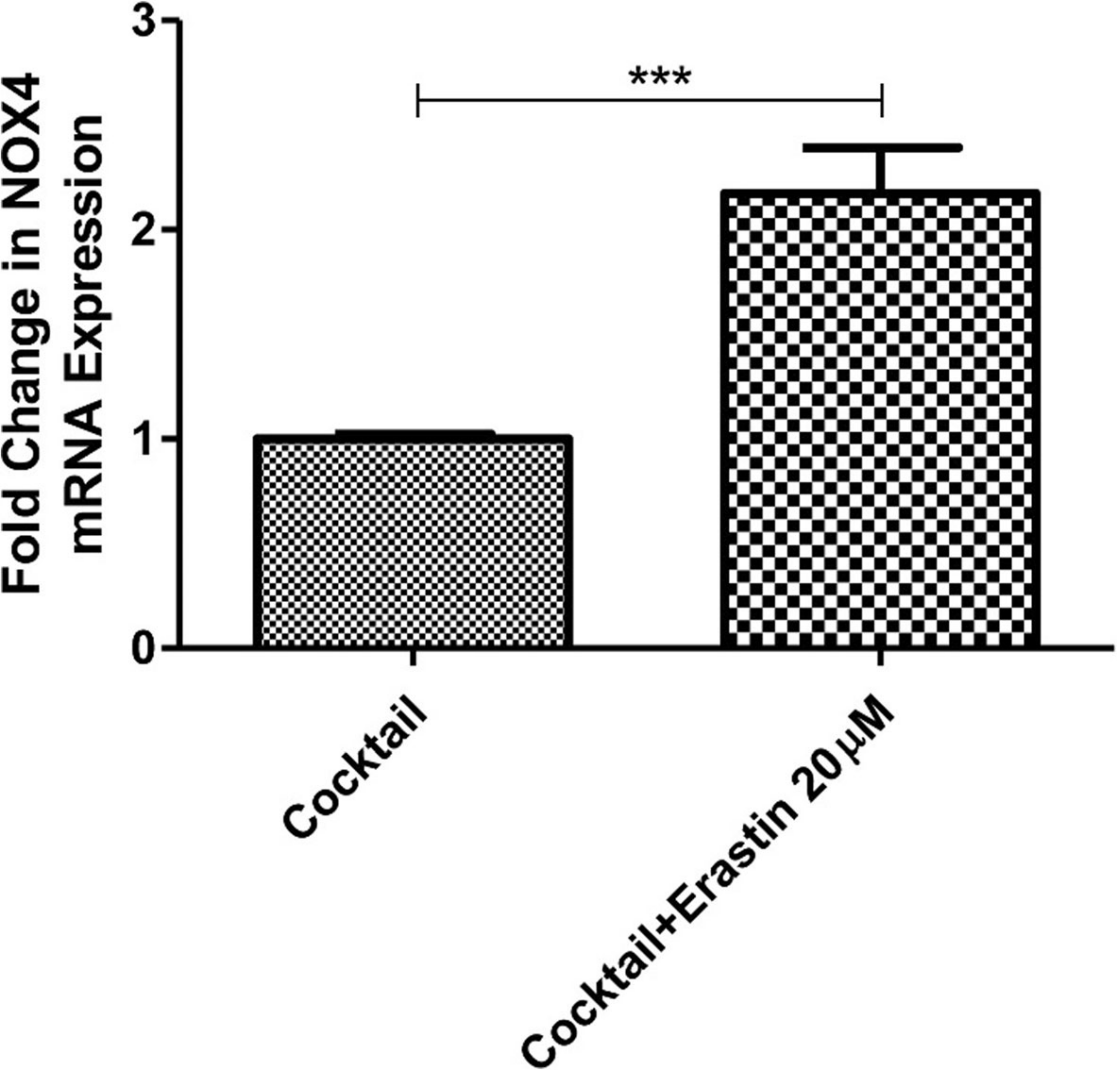
The change in gene expression of NOX4 in ICCs with ferroptosis induction. The ICCs were treated with or without the use of erastin (20 μM) for 48 h and then harvested for the detection of the gene expression of NOX4 by real-time PCR analysis. (*n* = 3 per group; *p < 0.05, **p < 0.01, ***p < 0.001. All data are expressed as means ± SEM)

Since the NADPH oxidase 4 (NOX4) is closely associated with the generation of ROS, the latter being a critical mediator for the initiation of ferroptosis, we thus sought to examine its expression changes in the erastin-induced ICCs ferroptosis. Preliminary results showed that the expression of NOX4 in ICCs was markedly increased after the exposure with erastin treatment, suggesting that the potential involvement of NOX4-mediated ROS production in ICCs ferroptosis (Fig. 4).

### Erastin-induced ICCs ferroptosis appears not to trigger apoptosis or autophagy

**Fig. 5:**
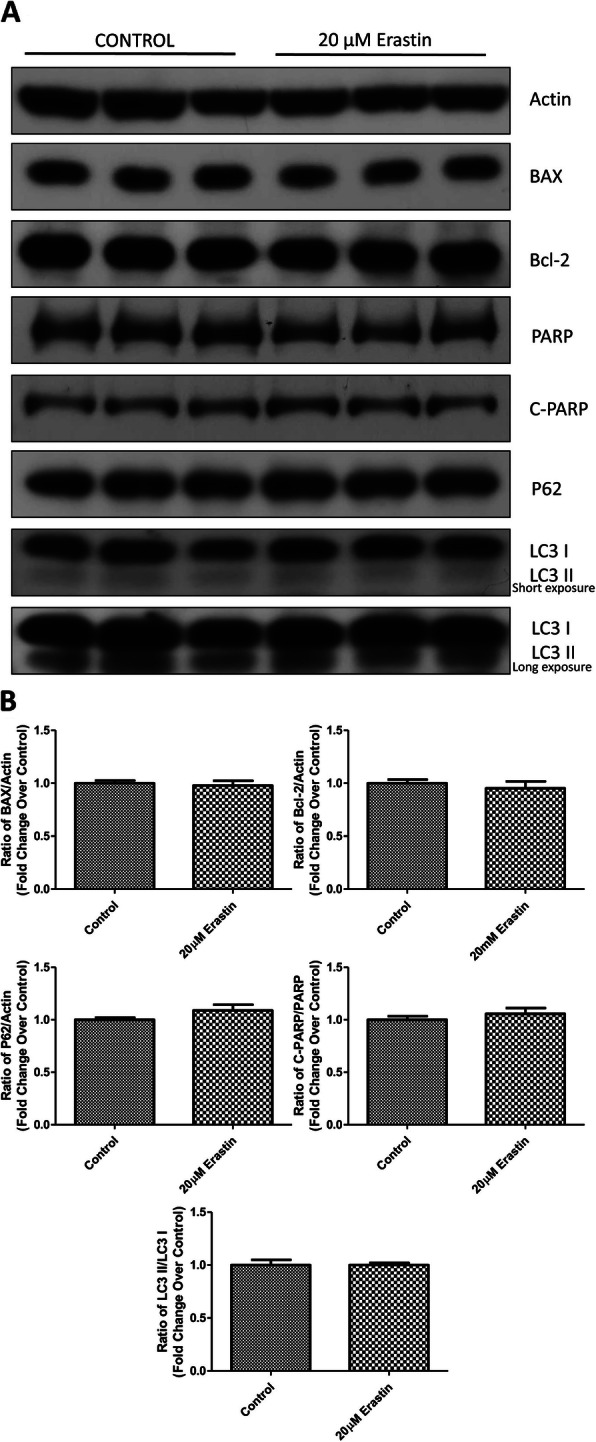
The assessment of other types of cell death potentially involved in erastin-induced ferroptosis in ICCs. The protein expression changes of the protein markers for apoptosis and autophagy were examined after ICCs were challenged by 20 μM erastin for 24 h. Western blot results with the blot images (**a**) and quantification (**b**) on the protein expression of BAX, Bcl-2, P62, PARP, cleaved PARP (C-PARP) and LC3 I/II as well as actin were performed and analyzed. (*n* = 3 per group; *p < 0.05, **p < 0.01, ***p < 0.001. All data are expressed as means ± SEM)

Given the existence of cross-talk between ferroptosis, apoptosis and autophagy, we further attempted to examine whether erastin-induced ferroptosis or other types of cell death was involved in our ICCs. Results from Western blot analysis showed that the protein expression profiles of commonly used markers for apoptosis (BAX, Bcl-2 and PARP) and for autophagy (P62 and LC3) were found to be unaltered after the challenge of ferroptosis inducer erastin (Fig. 5a & b). These preliminary data seem to propose that erastin-induced cell death can be a type of ferroptosis in nature, which may be independent from apoptotic or autophagic cell death.

## Discussion

In the practice of clinical islet cell transplantation in patients with diabetes, the accepted quality of graft functionality and survival rate are instrumental in the determination of the engraftment outcomes and long-term success rates. To achieve this goal, it depends heavily on numerous cytotoxic conditions associated with peri-transplant period as well as the life-long use of immunosuppressants (Shapiro et al. 2017). In this context, the current progress on the potential utility of pancreatic stem/progenitor cells-derived ICCs/islet cells for transplantation holds great promise with clinical merit and research interest (Leung and Ng 2013). Among these detrimental factors, the transplantability of ICCs are exposed to an array of cytotoxic conditions/factors during their preparation and pre−/post-transplantation procedures, thus leading to cell death and negative impacts on the engraftment function. They include, but are not limited to, the hypoxia, ischemia, inflammation, revascularization, immunoreaction and etc. (Kanak et al. 2014).

Despite being preliminary in the present study, our original data are the first to have reported that our ICCs are likely subjected to ferroptotic cell death rather than apoptotic and autophagic ones at least under our cell culture conditions, probably via the activation of JNK/P38/MAPK signaling and upregulation of NOX expression. In term of clinical and translational research interest, interventional strategies have long been putting on the inhibition of apoptosis during transplantation; for example, the inhibition of X-linked inhibitor of apoptosis protein (XIAP) was previously employed to reduce the apoptosis of islet cells, thereby improving the engraftment function (Emamaullee and Shapiro 2006). In this perspective, ferroptosis, a new form of regulated cell death, has been recently garnered attention with a great potential of clinical implications, notably cancer treatment (Dixon et al. 2012; Mou et al. 2019). If confirmed, leveraging the advantage for the small molecules/drugs with ferroptosis-inducing or inhibiting capacity could improve the cell function and prolong the cell survival of human PPCs derived ICCs in the context of clinical islet transplantation. To explore this possibility, we sought to assess the response of our ICCs to the drug, erastin, that induces ferroptosis. Interestingly, our ICCs were found to be very susceptible to erastin-induced ferroptosis with impaired insulin-producing function. It has been previously reported that erastin-mediated ferroptosis could trigger other type of cell death, depending on the cell types studied (Yu et al. 2015). In the present study, we could not observe other types of cell death, such as apoptosis and autophagy, suggesting that the erastin-induced ferroptotic cell death might be unique in our ICCs.

It has been shown that the MAPK signaling pathways are closely linked to ferroptosis; however, the precise pathways involved are thought to be cell specific (Xie et al. 2016). In our study, we found the erastin-induced ferroptosis in ICCs being mediated by the activation of JNK and P38 but not by ERK signaling. Meanwhile, NOX4 expression is well known to be the key player for the generation of ROS; in fact, it has been previously reported that NOX4 expression is also associated with the initiation of ferroptosis in different cells (Wang et al. 2018; Poursaitidis et al. 2017). Interestingly, the gene expression of NOX4 levels were markedly elevated subsequent to the treatment with erastin in the present study. This preliminary result points to the potential role of NOX4-induced ROS mediated ferroptosis in our ICCs; this finding appears to be consistent with our previous study showing that NOX4-dependent ROS promotes the proliferation and differentiation of PPCs into ICCs under both in vitro and in vivo conditions (Liang et al. 2016). Nevertheless, the detailed regulatory mechanism(s) involved in the ICCs ferroptosis-mediated graft function and survival warrant to be further investigated intensively using respective cell and animal models.

## Conclusions

We observed that our ICCs are susceptible to and specific for ferroptotic cell death, probably via the upregulation of JNK/P38 and NOX4 signaling pathways. Our study findings provide a scientific basis of ferroptosis inhibition as a potential for the amelioration of ICC survival and functionality during peri-transplant period. We speculate that these novel and original, albeit preliminary, data would open up new avenues for ferroptosis-driven agents/drugs mediated transplantable islets/ICCs in patients with diabetes.

## Data Availability

Yes
